# Resistance to Thyroid Hormone Beta or Thyroid-Stimulating Hormone (TSH)-Secreting Pituitary Adenoma: A Diagnostic Dilemma With a THRB Variant of Uncertain Significance

**DOI:** 10.7759/cureus.116246

**Published:** 2026-09-14

**Authors:** Ahmed Elhassan, Ahmed Mahgoub, Isra Ali

**Affiliations:** 1 Endocrinology Department, Mohammed Bin Rashid University of Medicine and Health Sciences, Dubai, ARE; 2 Internal Medicine, Tanta University Hospital, Tanta, EGY; 3 Biomedical Sciences, Mohammed Bin Rashid University of Medicine and Health Sciences, Dubai, ARE

**Keywords:** autoimmune thyroiditis, inappropriate tsh secretion, pituitary microadenoma, resistance to thyroid hormone beta, thrb gene mutation, tsh-secreting pituitary adenoma, variant of uncertain significance

## Abstract

Elevated thyroid hormone concentrations with nonsuppressed thyroid-stimulating hormone (TSH) present a challenging diagnostic problem. Resistance to thyroid hormone beta (RTHβ), analytical interference, and a TSH-secreting pituitary adenoma (TSHoma) are important considerations. We describe a woman with persistent elevation of free thyroxine (FT4) and nonsuppressed or elevated TSH for more than five years, autoimmune thyroiditis, a heterozygous THRB c.284G>C (p.Gly95Ala) variant classified as a variant of uncertain significance (VUS), and a 4.9 × 4.4 mm pituitary microadenoma. Human anti-mouse antibody testing was negative, and subsequent free alpha-subunit measurement was within the reference range. The biochemical phenotype was compatible with RTHβ, but the VUS could not establish a molecular diagnosis, while the pituitary lesion could not be assumed to be causal. This case highlights the risk of diagnostic anchoring when genomic and imaging abnormalities coexist and emphasizes integrating longitudinal biochemistry, assay evaluation, molecular classification, and pituitary assessment before diagnosing RTHβ or TSHoma.

## Introduction

Elevated FT4 and/or free triiodothyronine (FT3) with a nonsuppressed thyroid-stimulating hormone (TSH) concentration is an uncommon biochemical pattern that requires systematic evaluation. After medication effects and analytical interference are considered, resistance to thyroid hormone beta (RTHβ) and thyroid-stimulating hormone-secreting pituitary adenoma (TSHoma) are among the most important competing diagnoses [1,2]. RTHβ is usually caused by pathogenic variants in THRB and is characterized by reduced tissue responsiveness to thyroid hormone, often with few clinical manifestations despite abnormal thyroid function tests [1]. In contrast, TSHoma is a rare pituitary neuroendocrine tumor that causes autonomous TSH secretion and inappropriate stimulation of thyroid hormone production; diagnosis relies on biochemical and clinical evidence integrated with pituitary imaging rather than imaging alone [2].

Neither genetic testing nor pituitary imaging should be interpreted in isolation. A THRB variant of uncertain significance (VUS) does not establish RTHβ without supporting segregation or functional evidence, while small pituitary lesions may be incidental [1,2]. The coexistence of autoimmune thyroiditis can further complicate the interpretation of thyroid function tests [3]. The present report emphasizes the extended longitudinal biochemical phenotype and the subsequent identification of a pituitary microadenoma, which created an additional and clinically important diagnostic dilemma.

## Case presentation

A 27-year-old woman was evaluated in 2019 after discordant thyroid function tests were identified during fertility assessment. She had previously received intermittent levothyroxine for presumed hypothyroidism but discontinued treatment because she perceived no clinical benefit. She did not have a clinical picture of overt thyrotoxicosis, with no prominent palpitations, tremor, heat intolerance, or unexplained weight loss. Her family history included thyroid disease in a sibling.

On February 26, 2019, FT4 was 27.7 pmol/L (reference range, 12.0-22.0 pmol/L), FT3 was 6.2 pmol/L (reference range, 3.1-6.8 pmol/L), and TSH was 8.31 µIU/mL (reference range, 0.27-4.2 µIU/mL). Thyroid autoimmunity was documented, with thyroid peroxidase antibody 485.8 IU/mL (reference range, <34 IU/mL) and thyroglobulin antibody 202.4 IU/mL (reference range, <115 IU/mL).

Thyroid ultrasonography in March 2019 showed an average-sized gland with diffuse heterogeneous echotexture consistent with thyroiditis and multiple hypoechoic micronodules. A 13 × 7 mm posterior left-lobe nodule with an internal cystic component and peripheral vascularity was identified, without suspicious cervical lymphadenopathy.

Because the elevated FT4 was accompanied by nonsuppressed TSH, the initial diagnostic question was whether the discordance reflected analytical interference or altered thyroid hormone feedback. Human anti-mouse antibody (HAMA) testing was negative, reducing the likelihood of HAMA-mediated immunoassay interference, although this did not exclude other assay-related causes.

Molecular testing was then undertaken to evaluate RTHβ. According to the original molecular laboratory report, the coding exons and exon-intron boundaries of THRB were enriched using Roche/NimbleGen sequence-capture technology and analyzed by next-generation sequencing on an Illumina platform. A heterozygous NM_000461.4:c.284G>C substitution affecting the first base of exon 5 and resulting in p.Gly95Ala was identified. At the time of analysis, the variant was absent from the gnomAD population database and the laboratory's internal database and had not been documented in the literature or database search performed by the laboratory. Seven of 10 in silico prediction programs suggested a potentially deleterious amino acid effect, and three of seven splice-prediction programs suggested possible aberrant splicing. However, these computational findings were not sufficient to establish pathogenicity; in the absence of functional or segregation evidence, the laboratory classified the alteration as a VUS. Familial segregation analysis was recommended but was not available.

Serial thyroid function testing demonstrated persistent biochemical discordance for more than five years (Table 1). FT4 was above the reference interval on 12 of 13 measurements, reaching 30.1 pmol/L. TSH was elevated on all 13 measurements and ranged from 4.37 to 16.90 µIU/mL. FT3 was initially high-normal and was mildly elevated on two measurements in 2020. Autoimmune thyroid disease also persisted; in November 2023, thyroid peroxidase antibody was 565 IU/mL and thyroglobulin antibody was 305 IU/mL.

**Table 1:** Longitudinal thyroid function tests from 2019 to 2024 Reference ranges: FT4, 12.0-22.0 pmol/L; FT3, 3.1-6.8 pmol/L; TSH, 0.27-4.2 µIU/mL. ↑, above reference range; -, not available; TSH, thyroid-stimulating hormone

Date	FT4 (pmol/L)	FT3 (pmol/L)	TSH (µIU/mL)
26 Feb 2019	27.7 ↑	6.2	8.310 ↑
23 Jun 2019	23.4 ↑	5.8	12.432 ↑
17 Sep 2019	22.5 ↑	6.2	7.160 ↑
29 Apr 2020	25.3 ↑	7.1 ↑	10.700 ↑
4 Oct 2020	26.6 ↑	7.0 ↑	13.100 ↑
15 Oct 2020	24.4 ↑	-	16.900 ↑
7 Dec 2020	30.1 ↑	-	9.400 ↑
25 May 2021	22.5 ↑	6.1	7.170 ↑
27 Jul 2023	21.3	-	4.470 ↑
2 Nov 2023	27.3 ↑	-	4.370 ↑
8 Feb 2024	24.2 ↑	-	8.280 ↑
26 Feb 2024	28.1 ↑	-	11.700 ↑
3 Apr 2024	29.5 ↑	-	10.500 ↑

Because inappropriate TSH secretion persisted despite the RTHβ-compatible phenotype, the next diagnostic question was whether autonomous pituitary TSH secretion could be present. MRI of the sella on June 15, 2021, demonstrated a 4.9 × 4.4 mm focus of low T2 signal and reduced enhancement in the left anterior pituitary, interpreted as a pituitary microadenoma. The pituitary stalk was central, with no parasellar mass, cavernous sinus abnormality, or optic chiasmal involvement. Representative coronal post-contrast images are shown in Figure 1.

**Figure 1:**
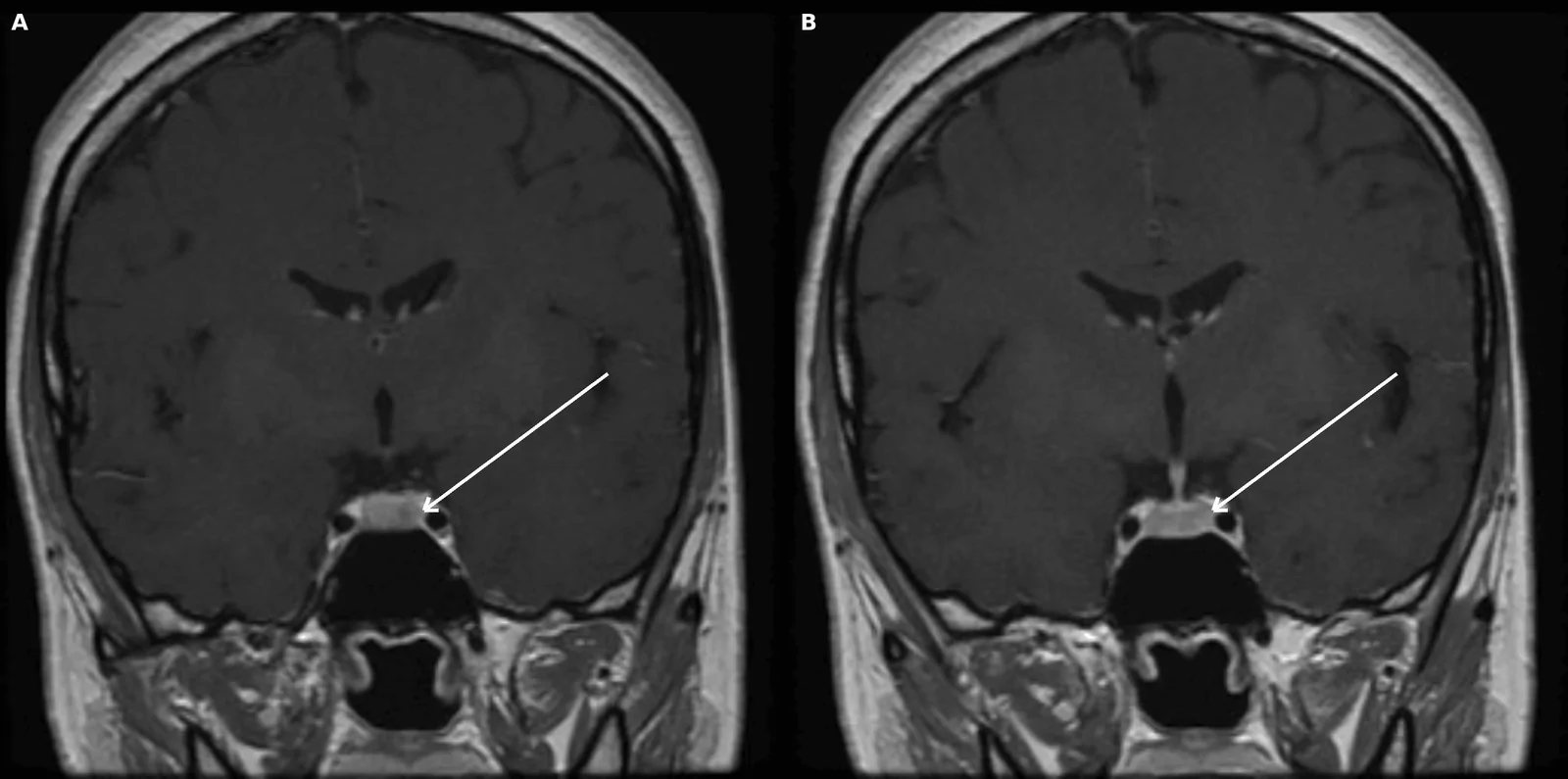
Coronal contrast-enhanced magnetic resonance imaging of the pituitary gland Representative coronal sellar images (A and B) from the contrast-enhanced pituitary MRI examination. Arrows indicate the left anterior pituitary region corresponding to the subtle area of relatively reduced enhancement identified on review of the complete dynamic examination. The focus measured approximately 4.9 × 4.4 mm and was reported as a pituitary microadenoma. The pituitary stalk was central, with no parasellar mass, cavernous sinus abnormality, or optic chiasmal involvement.

In April 2024, serum free alpha-subunit was 0.35 ng/mL (reference range, <1.02 ng/mL for premenopausal women). This did not provide biochemical support for a TSHoma, although a normal alpha-subunit was not considered sufficient to exclude a small TSH-secreting lesion in isolation. The patient remained clinically euthyroid and was managed conservatively with continued clinical and biochemical surveillance.

## Discussion

The central challenge in this case was determining the cause of persistent elevation of thyroid hormone concentrations with nonsuppressed TSH when two potentially relevant abnormalities were present: a THRB VUS and a pituitary microadenoma. The longitudinal biochemical pattern was strongly compatible with altered thyroid hormone feedback. RTHβ classically produces elevated thyroid hormone concentrations with nonsuppressed TSH, and affected individuals may remain clinically euthyroid [1]. Contemporary reviews emphasize that this biochemical pattern should trigger a structured evaluation rather than immediate attribution to either genetic resistance or pituitary disease [2,3].

However, the identification of a rare THRB alteration should not terminate the diagnostic process when that alteration remains a VUS. The c.284G>C substitution affects the first base of exon 5, and computational predictions raised the possibility of both an amino acid effect and abnormal splicing. These observations are supportive but not definitive. Current guidance recommends segregation of a THRB VUS with abnormal thyroid function in relatives when possible; if segregation cannot be established, dynamic assessment and functional studies can provide additional evidence [1,4].

The pituitary lesion complicated the interpretation further. MRI alone cannot establish a TSHoma because incidental pituitary abnormalities may occur in patients with RTHβ. In a large series of patients with discrepant thyroid function tests, 11 of 45 patients with RTHβ who underwent pituitary imaging had small pituitary lesions, while MRI was negative in some patients with genuine TSHomas [5]. Earlier reports likewise describe incidental pituitary adenomas or pituitary enlargement in patients with RTHβ, illustrating how imaging can misdirect the diagnosis [6,7]. Thus, attributing inappropriate TSH secretion to a small pituitary lesion without adequate biochemical corroboration risks diagnostic anchoring.

The normal free alpha-subunit concentration in this patient did not support TSHoma, but it does not definitively exclude a small TSH-secreting lesion. Conversely, the THRB VUS cannot independently confirm RTHβ. Guidelines and contemporary diagnostic reviews therefore favor a multimodal approach incorporating biochemical markers, genetic testing, dynamic endocrine testing when feasible, and pituitary imaging rather than relying on any single discriminator [1,8]. Recent reports also demonstrate that RTHβ and a functional TSHoma can rarely coexist, further reinforcing the need for integrated assessment [9].

Analytical interference remains an important consideration in any patient with discrepant thyroid function tests. Negative HAMA testing reduced the likelihood of one mechanism of interference but did not exclude other causes such as heterophile antibodies, anti-iodothyronine antibodies, abnormal binding proteins, or other assay-specific effects. A structured laboratory approach, including repeat measurement using an alternative assay architecture or a reference method when appropriate, can prevent unnecessary genetic or pituitary investigations [2,3,10]. In this retrospective case, detailed assay-platform information across the full five-year period and comprehensive cross-platform confirmation were not available; this limits the degree to which analytical interference can be excluded.

Autoimmune thyroiditis added another layer of complexity. Thyroid autoimmunity has been reported more frequently in patients with RTHβ than in unaffected relatives [11], and recent case literature continues to emphasize the diagnostic difficulty created when RTHβ coexists with autoimmune thyroid disease [12]. In this patient, strongly positive thyroid antibodies persisted over several years. Despite TSH concentrations reaching 16.90 µIU/mL, FT4 remained elevated rather than low, making uncomplicated primary autoimmune hypothyroidism an insufficient explanation for the overall biochemical pattern.

The THRB c.284G>C (p.Gly95Ala) variant was reported in 2024 in association with a biochemical phenotype suggestive of thyroid hormone resistance [13]. The clinical and biochemical features in that report overlap substantially with the early course described here. Accordingly, the present report does not claim novelty for the variant or for the initial RTHβ-like presentation. Its added educational value lies in the extended longitudinal biochemical course and the later diagnostic tension created by a persistent VUS together with a pituitary microadenoma. Published cases of RTHβ with incidental pituitary lesions, as well as rare coexistence of RTHβ and a functional TSHoma, demonstrate why neither imaging nor genetics should be interpreted in isolation [6,9,14].

Management of suspected RTHβ should be driven primarily by the clinical phenotype rather than an attempt to normalize circulating thyroid hormone concentrations [1,4]. Conversely, guidance for suspected TSHoma emphasizes biochemical and dynamic confirmation in conjunction with imaging before tumor-directed treatment [8]. The patient remained clinically euthyroid, and conservative surveillance avoided potentially inappropriate treatment while the etiology of the biochemical abnormality remained incompletely resolved.

This case is limited by the absence of familial segregation analysis, RNA or receptor functional studies, comprehensive cross-platform assay confirmation, detailed assay-platform information across the entire observation period, and dynamic endocrine testing. These limitations prevent definitive attribution of the phenotype to either the THRB variant or the pituitary lesion.

## Conclusions

Persistent elevation of thyroid hormone concentrations with nonsuppressed TSH requires careful integration of biochemical, genetic, and imaging findings. In this patient, a five-year RTHβ-like biochemical pattern coexisted with autoimmune thyroiditis, a THRB p.Gly95Ala VUS, and a pituitary microadenoma. Neither the genetic alteration nor the imaging finding independently established the diagnosis, and the available evidence did not definitively establish either RTHβ or TSHoma.

The case illustrates the importance of avoiding diagnostic anchoring when molecular and radiologic abnormalities coexist. Longitudinal biochemical assessment, systematic evaluation for analytical interference where feasible, appropriate interpretation of variant classification, and biochemical corroboration of pituitary findings are essential before attributing inappropriate TSH secretion to either RTHβ or a TSH-secreting pituitary tumor.
